# The Georgia Medical Association

**Published:** 1902-05

**Authors:** 


### EDITORIAL NOTES AND COMMENTS.

The Business office of THE JOURNAL-RECORD is No. 64 Marietta Street.

The Editorial office is 318-319-320 The Prudential.

Address all Business communications to Dr. M. B. Hutchins, Mgr.

Make remittances payable to THE ATLANTA JOURNAL-RECORD OF MEDICINE.

On matters pertaining to the Editorial and Original Communications address Dr. Bernard Wolff, Atlanta.

Reprints of original articles will be furnished at cost price. Requests for the same should always be made *on the manuscript*.

We will present, post-paid, on request, to each contributor of an original article, twenty (20) marked copies of THE JOURNAL-RECORD containing such article.

### THE GEORGIA MEDICAL ASSOCIATION.

THE fifty-third annual meeting of the Georgia Medical Association was held in Savannah on the 16th, 17th and 18th of April. It was one of the largest meetings in point of attendance in the history of the Association. This naturally was to be expected, since the meeting was held in Savannah, in this old Georgia city, far-famed for its hospitality and elegant entertainments. Since the large majority of the physicians of the State live exclusively inland, the desire to feel once more the breezes from over the ocean's depth is quite an incentive to many of them to make the trip. Then again the elegant hotel accommodations insured a comfortable time without crowding, and since the Medical Association is becoming such a large numerical body, it will soon have to meet in those cities only which can supply the necessary hotel facilities. The social features of the meeting were all that could be desired. On the first evening the Association enjoyed a delightful "smoker" given at the Savannah Yacht Club, where the presence of the ladies added much to the evening's entertainment. On Thursday evening a delightful fish and oyster dinner was given to the Association at the celebrated Thunderbolt hostelry. The menu card was exceedingly unique in character and was quite emblematic of many of the prescription party given by the physicians. The famous "artillery punch," although present, was as innocent as ever, but by means of its kindly sympathy much wit and humor was made to flow from the mouths of those who had gathered around the festal board. Among the notable toasts made upon this occasion must be mentioned those of Drs. Calhoun, Morgan, Baird and Graham.

On Friday afternoon an "oyster roast," given at Tybee, wound up the festivities of the meeting. This was a delightful affair and was enjoyed by both the ladies and gentlemen of the Association.

The program for this occasion was lengthy and showed a variety of subjects discussed. Many excellent papers were read, and some of them showed much research and study. On account of the length of the program, it was barely possible for all the papers to be read. If we might be permitted to make a wholesome suggestion, it is that in the future the time limit for discussions and the reading of papers be more closely adhered to and that the speakers be made to restrict their discussions to the subject in question.

The officers elected were Dr. Chas. Hicks, of Dublin, President, and Dr. R. D. Guinn, of Conyers, Vice-President.

The next meeting of the Association will occur in Columbus.

---

THE sympathy of the JOURNAL RECORD is extended to Dr. J. S. Todd in his bereavement at the death of his wife.

---

DR. W. H. MONCRIEF has passed the examination and received the appointment of assistant surgeon in the regular army.

